# Self-potential time series reveal emergent behavior in soil organic matter dynamics

**DOI:** 10.1038/s41598-022-17914-5

**Published:** 2022-08-08

**Authors:** Kennedy O. Doro, Nathan P. Stoikopoulos, Carl-Georg Bank, F. Grant Ferris

**Affiliations:** 1 Department of Environmental Sciences, University of Toledo, Toledo, OH USA; 2 Department of Earth Sciences, University of Toronto, Toronto, ON Canada

**Keywords:** Biogeochemistry, Environmental sciences, Geophysics

## Abstract

The active cycling of carbon between soil organic matter and the atmosphere is of critical importance to global climate change. An extensive body of research exists documenting the capricious nature of soil organic matter (SOM) dynamics, which is symptomatic of an intricate network of interactions between diverse groups of heterotrophic microorganisms, complex organic substrates, and highly variable local environmental conditions. These attributes are consistent with elements of complex system theory and the temporal evolution of otherwise unpredictable patterns of behavior that emerge from long range dependency on initial conditions. Here we show that vertical depth profile of self-potential (SP) time series measurements responds in a quantitative manner to variations in soil moisture, SOM concentrations, and relative rates of microbial activity. Application of detrended fluctuation analysis (DFA) of self potential time series data is shown additionally to reveal the presence of long-range dependence and emergence of anomalous electrochemical diffusion behavior, both of which diminish with depth as SOM specific energy densities decline.

## Introduction

The vast quantity of carbon stored as organic matter in the top 3 m of soils is greater than amounts that exist in the atmosphere and terrestrial vegetation combined^1–4^. Because of this, even a small change in the biogeochemical processes that contribute to the conversion of soil organic matter (SOM) into greenhouse gases such as carbon dioxide or methane could further exacerbate global climate change even as mean temperatures rise and extreme variations in rainfall become more frequent^5^. Higher turnover rates of SOM in surface soils and emission of greenhouse gases also threaten the vertical transport and long-term sequestration potential of organic carbon in deeper soil horizons where radiocarbon ages are commonly as old as one thousand to more than ten thousand years^1,4,6^. Investigation of these interconnected vulnerabilities in SOM dynamics has exposed significant challenges to researchers not only in terms of how to integrate traditional physical, chemical, and biological measurements in a quantitative manner, but also how to reduce the tremendous amount of time spent on sampling for multiple analyses. An alternative approach in soil hydrological and biogeochemical studies is the growing use of self-potential (SP) monitoring, a geophysical method based on passive measurement of electrical potential differences generated by the movement of charged ions and water through porous subsurface materials^7,8^.

SP has been used to locate ore deposits, map hydrothermal zones, monitor groundwater flow and characterize contaminated sites due to their associated electrochemical, thermoelectric, electrokinetic, piezoelectric, and redox effects^9^. The method holds considerable promise to be applied in new ways as demonstrated in this study, especially with respect to a time-series analysis of its signals which opens new opportunities for quantifying moisture dependent organic matter-microbial activity dynamics. In soils, natural electric potential gradients can be attributed to electrokinetic effects caused by flowing water and biochemical effects attributable to oxido-reduction phenomena^10^. Decoupling the source of SP signals is non-trivial but petrophysical relationship between SP signals and hydraulic gradient is well established through an electrokinetic coupling coefficient^9,11^. This approach facilitates signal interpretation by taking the coupled effects of mobile charged solutes, as well as the charged nature of stationary minerals and associated organic matter, into account^12^. SP signals therefore offer an indirect but robust approach to quantify soil processes including soil moisture, soil organic matter content and relative microbial activity driving biogeochemical changes in soils. In this study, we use strong correlations between measured SP signals and soil moisture, SOM, and relative microbial activity rates to demonstrate the capability of using SP to scale understanding of SOM dynamics in a non-invasive way with minimal disturbances. We also use a time-series analysis of SP data to demonstrate the transient behaviour of SOM-microbial activity rates.

## Results and discussion

### Soil hydraulic conditions and microbial activity

**Figure 1:**
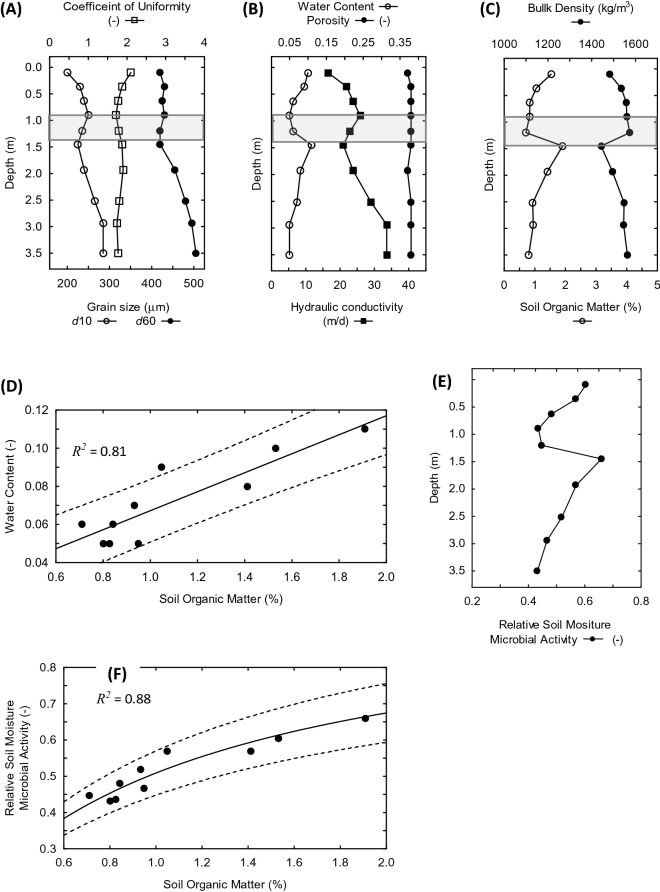
Soil depth profiles of hydraulic properties and relative levels of microbial metabolic activity. (**A**) Grain size distribution measurements of the tenth (*d*_10_) and sixtieth percentile (*d*_60_) particle diameters, along with the coefficient of uniformity $C_{u} = d_{60} /d_{10}$. (**B**) Calculated saturated hydraulic conductivities and porosities, as well as measured volumetric water content. (**C**) Soil organic matter contents estimated from measured bulk densities; the shaded interval (**A**)–(**C**) indicates the level where overall trends in hydraulic properties undergo a short reversal. (**D**) Volumetric water content as a function of soil organic matter. (**E**) Relative levels of soil microbial activity determined with respect to volumetric water content, and jointly with respect to relative amounts of microbial biomass. (**F**) Relative soil moisture microbial activities as a function of soil organic matter. The solid line represents the best fit of the Monod kinetics model for the dependence of relative soil moisture microbial activity under limiting amounts of soil organic matter. Dashed lines are prediction intervals at *p* values that represent the proportion of variance accounted for by the regression (*R*^2^).

The moderately high saturated hydraulic conductivity values determined for Meilleurs Bay terrace sands are conducive to rapid infiltration and downwards movement of meteoric precipitation through the unsaturated zone (Fig. 1A). While depth variations in hydraulic conductivities parallel corresponding fining and coarsening trends in grain size (Fig. 1A), the well-sorted nature of the sands constrain porosity to a narrow range near 39% (Fig. 1B). Measurements of bulk density follow the same trends, decreasing in the direction of fining upwards grain sizes and increasing with coarsening upwards grain sizes, with the lowest bulk densities close to the surface (1482 kg/m^3^) and 1.45 m underground (1443 kg/m^3^). Considering the nearly constant porosity and uniform mineral composition of the terrace sands, the observed variations in bulk density can be attributed directly to differences in soil organic matter content with the greatest amounts predicted for depths corresponding to the lowest bulk densities (Fig. 1C)^13–16^. The peak of SOM at 1.45 m depth is consistent with the presence of a root mat horizon in the upwards coarsening interval between 0.9 and 1.5 m depth^17^.

Post-wetting drainage dynamics of unsaturated porous media are complex owing to spatial and temporal changes in the extent of hydraulic continuity between pores^12,18,19^. At the time of this investigation, 24 h after a rainfall event, moisture levels were close to the residual volumetric water content anticipated for rapid drainage of meteoric precipitation through highly permeable sandy soils (Fig. 1B)^18^. Elevated water contents at 0.10 and 1.45 m depths correspond directly to depth levels with the smallest grain size, lowest hydraulic conductivity, and highest content of soil organic matter. Among these parameters, linear regression established that soil organic matter content accounted for the greatest proportion of the variance in volumetric water contents (*R*^*2*^ = 0.81; Fig. 1D). Similar relationships have been reported in other studies documenting the enhancement of water retention by soil organic matter^16,20^, including extracellular polymeric substances produced in situ by microorganisms^21^.

Decomposition of organic matter in soils by heterotrophic microbial respiration is strongly dependent on soil moisture^22,23^. This relationship is reflected in the similar depth profiles of calculated soil moisture microbial activity^24^ and measured volumetric water contents (Fig. 1E and B, respectively). Another underlying premise of the microbial activity-soil moisture function is that relative soil moisture microbial activity is reflective of the bioavailability of soil organic matter under partially saturated conditions^24^. This invokes the Monod kinetics model of microbial growth and substrate utilization under nutrient limiting conditions^22,25^. Recognizing that soil moisture and organic matter content are related (Fig. 1D) provides a useful paradigmatic link between the microbial activity-soil moisture function and bioavailability of organic matter implied by the Monod relationship. As shown in Fig. 1F, soil moisture microbial activity at different horizons across the depth profile is, in fact, responsive to organic matter concentrations, calculated independently as a function of bulk density. The implication is that soil moisture does limit the bioavailability of soil organic matter under fluctuating soil moisture conditions, with a half-saturation constant *K* = 0.97 + 0.03 for a relative soil moisture microbial activity equivalent to one-half of the level of microbial activity expected under optimal soil moisture conditions.

### Self-potential response

**Figure 2:**
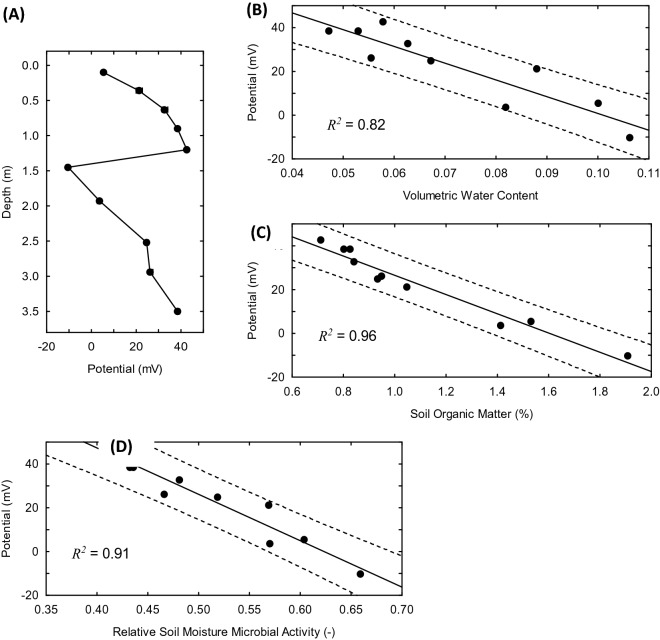
(**A**) Soil depth profile of mean self-potential values from 1200 s time series recorded at a sampling frequency of 1.0 Hz; the dot diameter indicating the mean values is larger than + 2 $\sigma$ of the time series. Self-potential response as a function of (**B**) volumetric water content, (**C**) soil organic matter content, and (**D**) soil moisture microbial activity; solid lines represent the best fits obtained for the corresponding linear regression equations. Dashed lines are prediction intervals at *p* values that represent the proportion of variance accounted for by the regression (*R*^2^).

Mean values of the SP time series (supplementary material Fig. S1), increased from 5.3 mV at 0.10 m to 42.7 mV at 1.20 m depth, then underwent a sharp decrease to − 10.4 mV at 1.45 m depth before resuming an upwards trend to 38.5 mV at 3.5 m (Fig. 2A). The trend reversal in SP response with depth is a mirror reflection of that observed with soil water content, soil organic matter, and soil moisture microbial activity. Of these parameters, water content accounts for 83% of the depth profile variation in SP (Fig. 2B) compared to 96% for soil organic matter (Fig. 2C) and 90% for soil moisture microbial activity (Fig. 2D).

The linear correlation between mean SP response and water content as an independent variable is consistent with numerous theoretical and experimental studies^9,11,12^; however, considerable variation exists in the reporting of coupling coefficients (i.e., slope of the regression), depending on the saturated streaming potential coupling coefficient ($C_{s}$) and how differences in pore water specific energy are expressed^10,26,27^. Here we apply a version of the Helmholtz-Smoluchowski equation for $C_{s}$ that incorporates the effective conductivity of a saturated porous medium, as defined by Archie’s Law^7,12,28,29^; relevant equations and parameters and are defined in the methods section. For an unsaturated porous medium, the difference in specific energy can be formulated in terms of volumetric water content as $dP = L_{c} \rho_{w} g\phi^{ - 1} d\theta$ where $L_{c}$ is a soil-specific characteristic length scale that defines the maximum extent of hydraulic continuity for gravity drainage^30^; $C_{s} L_{c} \rho_{w} g\phi^{ - 1}$ thus represents a conditional unsaturated streaming potential coefficient. For the field data, this approach gave -0.024 mV/Pa for $C_{s}$, a soil zeta potential of − 46.22 mV, and 0.10 for the volumetric water content ($\theta_{ref}$) at the shallow depth of the reference electrode. The first two values are consistent with those reported for fine quartz sands at low electrical conductivities (ca. 10^–3^ S/m) that accompany infiltration of dilute meteoric precipitation^26,31^, while the estimate for $\theta_{ref}$ is comparable to that measured at a depth of 0.10 m in the borehole (Fig. 1B).

The strong effect of SOM on SP is an intriguing finding that is related, in electrochemical terms, to surface charge development and cation exchange capacity of organic matter-mineral aggregates in soils, including those containing microbial biomass^12,32–34^. Recognition of the relationship between SOM and volumetric water content (Fig. 1D) allows for refinement of the streaming potential coupling coefficient and extends the theoretical basis for quantitative interpretation of variations in SP response to observed differences in soil organic matter concentrations (Fig. 2C). In this case, the difference in specific energy takes the form $dP = L_{c} \rho_{w} gm_{SOM} \phi^{ - 1} dSOM$ with $m_{SOM}$ equivalent to the slope of the linear regression of $\theta$ as a function of SOM; $L_{c}$ is a soil-specific length scale that defines the maximum extent of hydraulic continuity in the porous network as a function of parameters $\alpha$ and $n$ of the van Genuchten model of soil water retention^30^. The corresponding estimates for $C_{s}$ and soil zeta potential from the SP data are − 0.028 mV/Pa and − 55.2 mV, respectively. These estimates compare favorably to those derived on the basis of SP response as a function of volumetric water content. At the same time, a calculated reference electrode SOM concentration of 1.6% w/w is comparable to that at 0.10 m (1.53% w/w) and 1.45 m (1.41% w/w) depths in the borehole (Fig. 1C). As expected for this condition ($dSOM = \left[ {SOM} \right]_{ref} - \left[ {SOM} \right]_{borehole } \to 0$), the observed SP response at these depths is near 0 mV (Fig. 2A). This illustrates the potential to apply inverse modeling in SP surveys to map out SOM concentrations in soils by geophysical means.

The correlation between SP and soil moisture microbial activity presumably reflects the impact of changes in water content on mass transport processes and the manifest bioavailability of soil organic matter. Because optimal microbial activity is realized at a water saturation of 0.65^24,35^, the soil-specific characteristic length scale that defines the upper drainage limit at $$f_{SMA} = 1.0 $$ is taken to be $$0.65L_{c}$$^30^. This gives the difference in pore water specific energy in relation to microbial activity as $$dP = 0.65L_{c} \rho_{w} gdf_{h}$$, which yields estimates of -0.027 mV and -53.2 mV for $$C_{s}$$ and zeta potential, respectively. A predicted value of $$f_{h} = 0.62 $$ was ascertained for soil moisture microbial activity at the reference electrode, in good agreement those calculated from measured water contents at 0.10 m ($$f_{h} = 0.60)$$ and 1.45 m ($$f_{h} = 0.66)$$ depths in the borehole (Fig. 1E). Accounting for SP response as a function of soil moisture microbial activity through an unsaturated streaming potential coupling coefficient provides a new quantitative geophysical perspective on how changes in pore water energy status are apt to influence the bioavailability and fate of SOM in soils.

### Self-potential and long-range correlation

**Figure 3:**
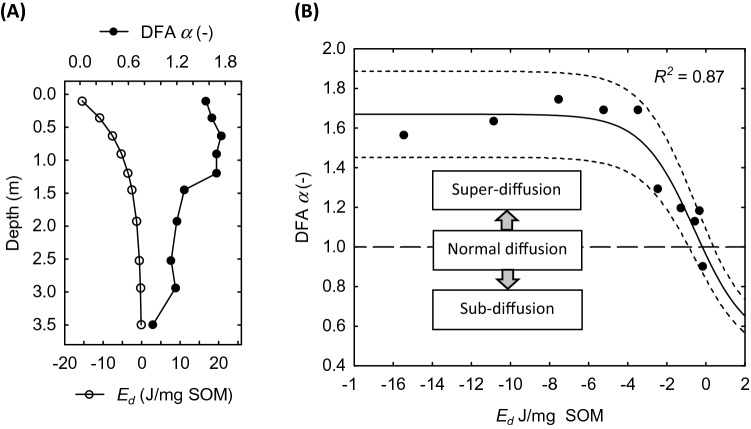
Soil depth profiles for (A) DFA α value scaling exponents with corresponding estimates for soil organic matter (SOM) energy density values, and (B) DFA α value scaling exponents as a function of soil organic matter (SOM) energy density values; dashed lines are prediction intervals at *p* values that represent the proportion of variance accounted for by the regression (*R*^2^).

The application of DFA to examine the correlation structure of time series data has become a widely used computational method in time series analysis^17,36–38^. The SP time series yielded DFA $\alpha$ scaling exponents that declined with depth from as high as 1.75 in the upper 1.45 m of the profile to 0.90 at 3.5 m depth (Fig. 3A). This trend reflects a progressive weakening of long-range correlation in temporal fluctuations of SP at deeper soil horizon levels^36,38,39^. As a signal dependent on the movement of water and ions in porous medium, measurements of SP contain information that pertains directly to underlying current flow and mass transport processes. Specifically, the local diffusive flux of each ionic species is described by the Nernst-Planck relationship in which ion mobility is determined by thermodynamic chemical potential gradients and migration in an electric field^12^. This is insightful for DFA of the SP time series because it points to diffusion of electrochemical energy as an important process in the manifestation of long-range correlations. Within this framework, the conventional model of Fickian diffusion applies in the case of classical Brownian motion where $\alpha$ = 1.0. Conversely, $\alpha \ne$ 1.0 denotes a family of anomalous diffusive behaviors that are described in terms of fractional Brownian noise as super-diffusion ($\alpha$ > 1.0) or sub-diffusion ($\alpha$ < 1.0), whereas $\alpha$ = 0.5 represents uncorrelated Gaussian white noise^40,41^. Extension of these concepts to the observed depth profile of DFA $\alpha$ values imply a transition from super-diffusion of electrochemical energy near the surface to weak sub-diffusion in deeper sub-soil horizons.

The most important nonequilibrium driver of energy flow in soil systems is organic matter. While SOM exhibits a high degree of heterogeneity with respect to molecular structure and biogeochemical reactivity, specific energy density (J/mg SOM) from differential scanning and bomb calorimetry measurements provide a direct estimate of the energy state of SOM with respect to susceptibility of oxidation to carbon dioxide. SOM specific energy density can be modeled as decreasing exponential function of depth based on measurements across a wide variety of soil types^42–47^ (Fig. 3A). This permits comparison of the SP time series DFA values with corresponding estimates of SOM energy densities (Fig. 3B). The trend evident in the data follows a distinctive pattern of a logistic transition between energy states with $\alpha$ values increasing as a function of SOM energy density^48^. The inflection point of the regression curve at -0.59 J/mg SOM is at an energy level where the predicted range of DFA $\alpha$ values evoke the onset of Brownian motion and normal diffusion in the SP fluctuations. Conversely, high SOM energy densities and $\alpha$ scaling exponents fall into the range of super-diffusive electrochemical behavior that is symptomatic of contributions from high rates of microbial respiration^37,49^. Moreover, a cross-over from super-diffusion ($\alpha$ > 1.0) to sub-diffusion ($\alpha$ < 1.0) is apparent across a narrow range of SOM energy densities (− 2.0 to − 4.0 J/mg SOM) that implies a transition from non-equilibrium microbial catalyzed to mass action controlled chemical reactivity (e.g., mineral-SOM interactions) as the primary determinant of electrochemical energy flow in deeper subsoil horizons^48,49^.

### Emergent behavior in soil organic matter dynamics

There is ample evidence supportive to extending the paradigm of complex system theory to SOM dynamics^3,32,50^. Recognition of a high degree of spatiotemporal variation of interactions between physical, chemical, and microbial processes under non-equilibrium conditions in soils is an especially compelling consideration. Another hallmark of complex systems is the manifestation of emergent behavior in the form of a perceptible degree of long-range dependence and sensitivity to initial conditions within a rich network of interacting processes^5,6,51,52^. These attributes are mirrored not only by the observed correlations of self potential to soil moisture, SOM content and microbial activity (Fig. 2), but also the detection of long-range dependency and emergence of anomalous diffusion behavior in the SP time series data. Moreover, the progressive loss of long-range dependence with depth and approach to normal diffusion behavior is consistent with a weakening and possible loss of some network interactions with declining SOM energy densities.

Another insightful aspect of the logistic relationship between DFA $\alpha$ values of the SP time series and SOM energy density is the changing width of the nonlinear regression prediction interval (Fig. 3B). Of particular interest is the greater range of the prediction interval ($\alpha \in \left[ {1.45, 1.90} \right])$ at energy densities $\le$ -3.0 J/mg SOM, which is representative of shallow depths $\le$ 1.20 m. Considering that $\alpha$ depicts the strength of long-range dependence and emergence of anomalous diffusivity, the wide prediction interval suggests that potentially small perturbations in the underlying network of interacting dynamic variables (e.g., soil moisture, SOM content, microbial activity) could trigger almost an order of magnitude shift in emergent behavior. The potential for such unpredictable “chaotic” changes is perceived as another characteristic feature of temporal dynamics in complex systems, including heterogeneous soil environments^5,6,53^. Conversely, the much-narrowed prediction interval at SOM energy densities $\ge$ − 2.0 J/mg SOM signifies a weakening of long-range dependency and dissipation of anomalous diffusion behavior owing to a fragmentation of network interactions.

### Conclusion

The correlation between SP and SOM, soil moisture and soil microbial activity demonstrated in this study highlights a new opportunity to quantify soil organic matter and soil moisture-microbial activity. The complexity with direct measurements of these soil properties makes alternative approaches valuable for constraining and scaling up the spatial and temporal resolution of their estimates. While this study uses borehole SP measurements, surface measurement of SP has been demonstrated for groundwater and contaminated sites characterization. Hence, SP provides a non to minimally invasive geophysical tool which when combined with minimal direct measurement would improve the characterization and monitoring the spatial and temporal variations in soil organic matter and microbial activity. This is bound to expand our mechanistic understanding of the capricious nature of soil organic matter. Besides elucidating the transient nature of electrochemical energy flow in SOM relatable to soil microbial respiration, the time-series SP data acquisition presented in the studies challenges the conventional approach of electrical geophysical data acquisition broadly applied in subsurface characterization and monitoring including complex environmental systems^9,10,12,38^. Specifically, electrical signatures are commonly measured multiple times and an average value calculated and attributed to a given location. In complex and emergent system, this ignores the pronounced temporality that may be inherent in such system. We therefore recommend adoption of a time-series measurement approach for soil electrical signals (mostly natural potentials) as this is apt to capture temporal perturbations indicative of complex time-varying emergent processes within the subsurface.

## Methods

### Study site description

The study site is a small forest clearing (NAD 83 UTM coordinates 18 T 297593E 5115217 N; elevation 133 masl) in the upper reaches of the Meilleurs Bay catchment on the south bank of the Ottawa River, approximately 10 km northwest of Deep River, Ontario, Canada^13^. The mean annual temperature in the region is 4.9° C, ranging from 18.4 °C in July to − 10.5 °C in January^54^. The average annual precipitation is 878 mm with the local climate falling into the Köppen classification scheme as Dfb, humid continental^55–57^. The forest at the study site is composed of balsam fir (*Abies balsamea*), red maple (*Acer rubrum*), yellow birch (*Betula alleghaniensis*), white spruce (*Picea glauca*), eastern white pine (*Pinus strobas*), and poplar (*Populus tremuloides*). Prominent species along the edge of the clearing are wild blueberry (*Vaccinium angustifolium*), black raspberry (*Rubus occidentalis*), and wild strawberry (*Frangaria vesca*). Ground vegetation in the clearing includes fescues and tufted grasses (*Festuca* and *Lolium* sp.), goldenrod (*Solidago* sp.), sweet fern (*Comptonia peregrina*) and hawkweed (*Hieracium* sp.).

Around 10.5 ka BP, the retreat of the Laurentide Icesheet from the Precambrian Canadian Shield opened the Ottawa River valley to drainage from pro-glacial meltwater lakes in the Huron and Michigan sub-basins of the Great Lakes^13,58^. This discharge contributed to extensive deposition of medium- to fine-grained glaciofluvial channel sands along the slopes of the river valley. As water levels fell in response to incremental icesheet recession and changes in drainage routes of the pro-glacial lakes, subaerial exposure of the channel sand deposits contributed to the development of humo-ferrric podzol soils (Canadian soil classification scheme), which dominate over much of the upper Ottawa Valley region, including the Meilleurs Bay locality^4,17^. At the Meilleurs Bay, a series of terraces between 129 and 141 masl document brief fluctuations in river height during the recession of the Laurentide Ice Sheet^36^.

Instrumentation of the Meilleurs Bay catchment with an extensive network of piezometers has established that the unsaturated vadose zone at the study site extends to a depth of 5.0 m or more^59^. Seasonal and interannual fluctuations in water table depths throughout the aquifer are small, typically less than $\pm$ 0.5 m. Regional hydrological studies indicate that approximately 37 percent of the annual precipitation contributes to groundwater recharge or runoff. The remainder is returned directly to the atmosphere by evapotranspiration. Almost all the groundwater recharge occurs during and just after the spring snowmelt, whereas strong evapotranspiration during the summer months gives rise to soil moisture deficits.

### Soil depth profile and self-potential measurements

An open-barrel stainless steel sand auger with a diameter of 5 cm was used to advance a borehole to a final depth of 3.5 m through the unsaturated vadose zone at the study site. Sediment samples were collected for determination of grain size distribution, bulk density, and volumetric water content at 0.15 to 0.50 m intervals. Sample temperatures were recorded immediately after collection using a Solinst Levelogger to facilitate estimation of prevailing water density ($\rho_{w}$) and dynamic viscosity ($\mu$) values. Saturated hydraulic conductivities were calculated from the grain size distributions using a modified Hazen equation^60^

1 $$ K = 1.52 \times 10^{ - 3} \left( {\frac{{\rho_{w} g}}{\mu }} \right) C_{u} \left( {d_{60} } \right)^{2} $$

with the coefficient of uniformity ($C_{u}$) equal to the ratio between the 60th ($d_{60}$) and 10th ($d_{10}$) percentile particle size diameters (in meters) and $g$ corresponding to the acceleration of gravity. Porosity ($\phi$) was calculated as a function of $C_{u}$^24,47^.

2 $$ \phi = 0.2\left( {1 + 0.93^{{C_{u} }} } \right) $$

Bulk densities ($\rho_{b}$) were used to estimate soil organic matter concentrations (SOM % by weight) following the relationship derived for sandy forest soils of glacial fluvial origin^10,26^

3 $$ SOM = - 7.30 ln\left( {\frac{{\rho_{b} - 0.70}}{0.95}} \right) $$

Measurements of self potential were made at each depth interval using a Fluke 289 logging multimeter with an internal impedance of 20 MΩ. A pair of Cu–CuSO_4_ non-polarizing electrodes were connected to the terminals of the multimeter by two 30 m lengths of 18 gauge solid-core insulated copper wire. The lead electrode was fastened to a length of 2.5 cm outer diameter PVC conduit (to permit installation and retrieval from the borehole) and connected to the positive terminal of the multimeter. The reference electrode was connected to the negative terminal and inserted 0.15 m into the ground approximately 40 m away from the borehole. Readings were recorded as a time series at a frequency of 1 Hz over an interval of 1200 s (20 min). Potential differences recorded by the multimeter represent an average of five readings taken over the course of one second.

### Assessment of microbial activity

Relative rates of microbial heterotrophic respiration were calculated as a function of volumetric water contents using the microscale soil moisture microbial activity function ($f_{h}$) developed by Yan et al.^24^

4 $$ f_{h} = \frac{{r_{\theta } }}{{r_{max} }} = \left( {\frac{{K_{\theta } + \theta_{opt} }}{{K_{\theta } + \theta }}} \right)\left( {\frac{\theta }{{\theta_{opt} }}} \right)^{1 + an} $$

The value of $$f_{h}$$ compares heterotrophic respiration rates ($$r_{\theta }$$) at different levels of soil moisture ($$\theta$$) to the maximum rate ($$r_{max}$$) sustained by an optimal water content ($$\theta_{opt}$$), which is taken to be approximately 65 percent of soil porosity ($$\phi$$) (i.e., $$\theta_{opt} = 0.65\phi$$). Suggested values of $$K_{\theta }$$ = 0.1 for the soil organic matter desorption constant and $$n$$ = 2 for the saturation exponent were used along with the soil organic matter-microorganism collocation factor $$a$$, estimated as a function of fine grain mineral content (< 45 µm diameter fraction; $$a = 2.8c_{45} - 0.046 $$), to complete parameterization of the $$f_{h}$$ function^24^.

At soil moisture values below $0.65\phi$, the loss of hydraulic continuity between pores restricts soil organic matter bioavailability and limits heterotrophic microbial activity as predicted by the Monod microbial growth model^24,25^.

5 $$ r_{\theta } = \gamma \mu_{max} m \frac{{\left[ {SOM} \right]}}{{K_{S} + \left[ {SOM} \right]}} = r_{max} \frac{{\left[ {SOM} \right]}}{{K_{S} + \left[ {SOM} \right]}} $$

Rearrangement yields

6 $$ f_{h} = \frac{{r_{\theta } }}{{r_{max} }} = \frac{{\left[ {SOM} \right]}}{{K_{S} + \left[ {SOM} \right]}} $$

Here $\gamma$ is the yield coefficient for microbial growth on soil organic matter, $\mu_{max}$ is the maximum microbial growth rate constant, $m$ is the microbial biomass concentration, $\left[ {SOM} \right]$ is the soil organic matter concentration (%), estimated from bulk density values, and $K_{S}$ is the half-saturation constant (i.e., the SOM concentration where the rate of heterotrophic respiration is 0.5 $r_{max}$).

### Evaluation of self-potential response

In a saturated porous medium, the difference in self-potential $\varphi$ with respect to specific energy of pore water, expressed in terms of pressure ($P$, energy per unit volume) is proportional to a streaming potential coupling coefficient $C_{s}$

7 $$ - \frac{\partial \varphi }{{\partial P}} = C_{s} $$

The Helmholtz-Smoluchowski equation gives $C_{s}$ as a function of the electrical permittivity of water ($\epsilon$), zeta potential ($\zeta$), dynamic viscosity ($\mu$), and effective conductivity ($\sigma$)

8 $$ C_{s} = \frac{\epsilon \zeta }{{\mu \sigma }} $$

The influence of pore space connectivity on effective conductivity is described by Archie’s Law

9 $$ \sigma = \sigma_{w} \phi^{{\mathfrak{m}}} $$

where $\sigma_{w}$ is the electrical conductivity of the water, $\phi$ is porosity, and ${\mathfrak{m}}$ is the porosity (or cementation) exponent^12,28^. This gives

10 $$ C_{s} = \frac{\epsilon \zeta }{{\mu \sigma_{w} \phi^{{\mathfrak{m}}} }} $$

As water saturation ($S_{w} = \theta /\phi$) decreases during drainage, the change in pore water specific energy is characterized by a soil-specific length scale ($L_{c}$) that defines the maximum extent of hydraulic continuity in the porous network as a function of parameters $\alpha$ and $n$ of the van Genuchten model of soil water retention

11 $$ L_{c} = \frac{1}{{\alpha \left( {n - 1} \right)}}\left( {\frac{2n - 1}{n}} \right)^{{\frac{2n - 1}{n}}} \left( {\frac{n - 1}{n}} \right)^{{\frac{1 - n}{n}}} $$

Within the limits of drainage between saturated and residual water contents of a soil

12 $$ \frac{\partial P}{{\partial S_{w} }} = \phi \frac{\partial P}{{\partial \theta }} = L_{c} \rho_{w} g $$

Rearrangement and substitution into (7) yields

13 $$ - \frac{\partial \varphi }{{\partial P}} = - \frac{\partial \varphi }{{\partial \theta }} = C_{s} \frac{{L_{c} \rho_{w} g}}{\phi } $$

which gives a linear relationship for self-potential differences as a function of volumetric water contents at reference ($\theta_{ref}$) and measurement ($\theta$) electrodes.

14 $$ \partial \varphi = C_{s} \frac{{L_{c} \rho_{w} g}}{\phi }\theta_{ref} - C_{s} \frac{{L_{c} \rho_{w} g}}{\phi }\theta $$

Estimates for $C_{s}$ (mV/Pa), $\zeta$ (mV), and $\theta_{ref}$ ( −) were calculated from the slope and intercept of the regression of field data (SP as a function of $\theta$) using values reported for ${\mathfrak{m}} = 1.3$ ( −)^12^, $\alpha = 14.5$ (m^-1^) and $n = 2.68$ ( −)^61^ together with porosity from grain size analysis ($\phi = 0.37)$.

### Energy density of soil organic matter

The energy density ($E_{d}$) of soil organic matter tends to decrease in an exponential manner with respect to depth $z$^43^,

15 $$ E_{d \left( z \right)} = E_{{d \left( {z = 0} \right)}} exp - kz $$

with a decay constant $k$ = 1.35 + 0.19 m-1, and surface $E_{{d \left( {z = 0} \right)}}$ =  − 17.70 + 0.60 J/mg SOM ascertained for 125 measurements of SOM $E_{d}$ at different depths across a wide variety of soil types^42–47^. This empirical relationship was applied to derive estimates for SOM $E_{d}$ values at borehole depths corresponding to each of the measured SP time series.

### Data analyses

Nonlinear estimation using Levenberg–Marquardt optimization in STATISTICA 13.2 was employed to derive parameter estimates and the portion of variance accounted ($R^{2}$) for in dependent variables of regression equations. Analyses of the self-potential time series were performed in Python (3.8) by detrended fluctuation analysis (DFA) using the dynamical systems package Nolds (0.5.2) following the procedure described by Peng et al.^27^; the minimum time interval of windows was limited to 60 s in length, and computations were performed using both non-overlaping windows and successsive windows overlapped by half of their length. To further refine our estimate of DFA precision, each time series was downsampled into two time series equivalent to a sampling frequency of 0.5 Hz and the scaling exponents of the two new time series were calculated independently as described above. For all of the time series, the standard error of mean DFA $\alpha$ values was $< \pm$ 0.06. Random shuffling of each time series yielded $\alpha$ = 0.5, as expected for an uncorrelated signal.

## Supplementary Information

Supplementary Information.

## Data Availability

Data available on request from the corresponding author.
