# Supplementary material for: Self-potential time series reveal emergent behavior in soil organic matter dynamics
Source: Sci Rep. 2022 Aug 8;12:13531. doi: 10.1038/s41598-022-17914-5 (PMC9360037; doi:10.1038/s41598-022-17914-5)

**Self-Potential Time Series Reveal Emergent Behavior in Soil Organic Matter Dynamics**

Kennedy O. Doro[1,*], Nathan P. Stoikopoulos[2], Carl-Georg Bank[2], F. Grant Ferris[2]

1. Department of Environmental Sciences, University of Toledo, OH, U.S.A.

2. Department of Earth Sciences, University of Toronto, Toronto, ON, Canada

* kennedy.doro@utoledo.edu; +1 419 322 5283

**Supplementary Materials**

Table S1: Regression equations and parameter estimates for lines of best fit in data plots shown in figures 1 through 3.

| Regression Equations | Parmeter Estimates (± Standard Error) |
|---|---|
| Volumetric water content as function of soil organic matter (SOM %) $$\theta_w = b + m * SOM$$ | $b$ = 0.019 ± 0.009<br>$m$ = 0.048 ± 0.008 |
| Relative soil moisture microbial activity (*SMMA*) as function of soil organic matter (SOM %) $$f_h = \frac{SOM}{K_S + SOM}$$ | $K_S$ = 0.97 ± 0.03 |
| Self potential (mV) as a function of volumetric water content (-) $$SP = b + m * \theta_w$$ | $b$ = 77.38 ± 3.87<br>$m$ = -765.87 + 51.83 |
| Self potential (mV) as a function of soil organic matter (SOM %) $$SP = b + m * SOM$$ | $b$ = 70.46 ± 1.57<br>$m$ = -43.94 ± 1.35 |
| =Self potential as a function of relative soil moisture microbial activity ($f_h$) $$SP = b + m * f_h$$ | $b$ = 132.16 ± 5.18<br>$m$ = -211.97 ± 9.89 |
| Detrended fluctuation analysis $\alpha$ value as a function of SOM energy density $$\alpha_E = \alpha_{min} + \frac{(\alpha_{max} - \alpha_{min})}{1 + \exp\left(\beta[E - E_\beta]\right)}$$ | $\alpha_{min}$ = 0.51 + 0.09<br>$\alpha_{max}$ = 1.68 + 0.09<br>$\beta$ = 0.72 + 0.26 mg/J<br>$E_\beta$ = -0.59 + 0.19 J/mg SOM |
| SOM specific energy density $$\frac{dE}{dz} = -kz$$ $$E_z = E_{z=0}\, exp - kz$$ | $E_{z=0}$ = - 17.70 + 0.60 J/mg SOM<br>$k$ = 1.35 + 0.19 $m^{-1}$ |

Figure S1: Measured self-potential time series for depth intervals (A) 0.10 to 1.20 m, and (B) 1.45 to 3.5 m.

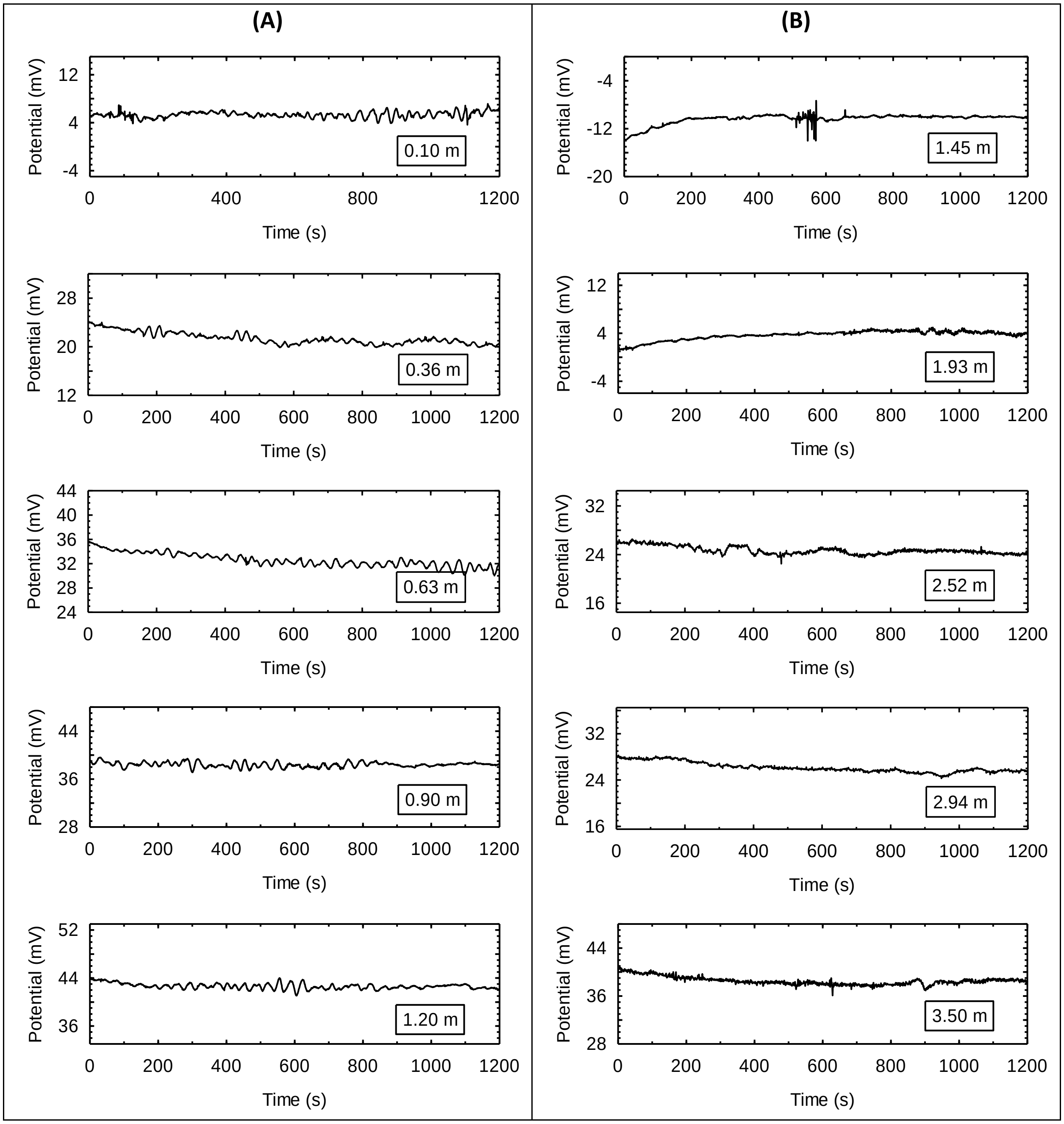

Supplement: Supplementary file 1 — Supplementary Information. [file 41598_2022_17914_MOESM1_ESM.pdf]
